# Community perceptions of the socio-economic structural context influencing HIV and TB risk, prevention and treatment in a high prevalence area in the era of antiretroviral therapy

**DOI:** 10.2989/16085906.2017.1415214

**Published:** 2018-03

**Authors:** Nothando Ngwenya, Dumile Gumede, Maryam Shahmanesh, Nuala McGrath, Alison Grant, Janet Seeley

**Affiliations:** 1 Africa Health Research Institute, KwaZulu-Natal, South Africa; 2 Africa Health Research Institute, School of Nursing and Public Health, University of KwaZulu-Natal, Durban, South Africa; 3 Institute of Global Health, University College London, London, UK; 4 Faculty of Medicine and Faculty of Social, Human and Mathematical Sciences, Southampton University, Southampton, UK; 5 London School of Hygiene & Tropical Medicine, London, UK; 6 School of Public Health, University of the Witwatersrand, Johannesburg, South Africa; 7 Research Department of Epidemiology & Public Health, University College London, London, UK

**Keywords:** community cohesion, efficacy, inequity, perceived control, social mobility

## Abstract

Following calls for targeted HIV prevention interventions in so-called “hotspots”, we explored subjective perceptions of community members in places considered to be high HIV and tuberculosis (TB) transmission areas and those with low prevalence. Although more people now have access to antiretroviral therapy (ART), some areas are still experiencing high HIV transmission rates, presenting a barrier to the elimination of HIV. A rapid qualitative assessment approach was used to access a sample of 230 people who contributed narratives of their experiences and perceptions of transmission, treatment and prevention of HIV and TB in their communities. Theoretical propositions case study strategy was used to inform and guide the thematic analysis of the data with Research Department of Epidemiology & Public Health, University College London, London, UK. Our results support the concept of linking perceived control to health through the identification of structural factors that increase communities’ sense of agency. People in these communities did not feel they had the efficacy to effect change in their milieu. The few socio-economic opportunities promote social mobility in search of better prospects which may have a negative impact on community cohesion and prevention strategies. Communities were more concerned with improving their immediate social and economic situations and prioritised this above the prevention messages. Therefore approaches that focus on changing the structural and environmental barriers to prevention may increase people’s perceived control. Multifaceted strategies that address the identified constructs of perceived control may influence the social change necessary to make structural interventions successful.

## Introduction

The place, or community, where people live or work, has an impact on their lives and health outcomes (Brooks et al., 2014; Goodwin & Engstrom, 2002). The perception of that place, held by the individual or by outsiders, is central to a person’s experiences, expectations, motivations and behaviour (Kalali, 2015) and plays a role in how people develop and describe their identity as well as that of their community. Therefore understanding these perceptions may be integral to adapting complex interventions to the context (MRC, 2006).

The drive to make public health policy more relevant and evidence based, necessitates research into individuals’ experiences and perceptions to investigate how places influence health and health-related behaviours. The influence on health includes the way people perceive the social quality of a place, such as cleanliness, neighbourhood cohesion, housing, employment, and access to health care (Ellaway, Macintyre, & Kearns, 2001). The labelling of a place by insiders and outsiders, involves aspects of power which influence people’s identity and behaviour affecting self-esteem, confidence and perceived sense of control (Kalungu-Banda, 2008). The label “hotspot”, for example, has been used in different ways in the context of health and disease to describe the type of place where disease transmission occurs (Alfaki, Salih, Elhuda, & Egail, 2016; Kupferschmidt, 2015). The HIV prevention domain suggests that such locations, whether a geographical space or a type of place, offer the opportunity to target interventions to those most at risk of HIV infection living or working in those locations (Tanser, de Oliveira, Maheu-Giroux, & Bärnighausen, 2014). However, an awareness of the effect of this label, operational in academic and at times policy discourse, may have an impact on individuals’ perceptions and aspirations (Collyer, 2006). It is from these perceptions that behaviours can be developed, influencing the perceived sense of control that community members have over the progress of the epidemic.

### Control, empowerment and efficacy

Perceptions of interest in developing solutions for pressing public health issues involve the belief in people’s capability to influence anticipated results. Evidence exists of a positive correlation between perceived control and positive health outcomes (Andrade & Devlin, 2016; Lachman & Weaver, 1998; Rodin, 1986; Thompson & Spacapan, 1991). Although largely influenced by past occurrences and events, perceived control is a good predictor of future health behaviour and outcomes (Bandura, 1982; Meyer-Weitz, 2005; Wallston, Wallston, Smith, & Dobbins, 1987). It encompasses the extent to which people believe they have control over their living situation and environment to effect the change needed to improve wellbeing. The control that an individual has depends on their behaviour and that of the people around them (Rotter, 1975). Therefore the networks one has, and the value the members put on health, have an impact on perceived control.

### Connectedness, social networks and community cohesion

Social networks can facilitate the spread of disease (Berkman, Glass, Brissette, & Seeman, 2000; Klovdahl et al., 2001; Zelner et al., 2012). However, cohesion within a community can be a marker of empowerment which is key to developing structural health interventions to address disease spread and poor health (Argento et al., 2016; Fonner et al., 2014). This is consistent with previous research which has shown social cohesion to be a determinant of health (Mulvaney-Day, Alegría, & Sribney, 2007) and a tool in HIV prevention (Kerrigan et al., 2013; Skovdal & Ogutu, 2012). Fostering support through social networks can help solve problems within a community, through identifying factors and properties of networks that garner support for change and highlight drivers of, for example, the HIV epidemic.

The relationships between people and context may not be easily captured using quantitative methods (Mitchell, 2001). We used multiple qualitative methods to capture and generate insights into processes of how people relate to their place and the link to health outcomes. This paper addresses a public health problem: the continued high incidence of HIV and TB through identifying structural or environmental factors which are perceived to influence behaviour in particular geographical communities.

### “Uvo Lwakho” meaning “your perception”

The process of finding a title for the study shed light on people’s views about the place they lived. A team of qualitative interviewers was recruited from the study area and worked with the lead investigator to find a name for the study which would be easily understood by those taking part, since the title appears on information sheets given out to participants. The isiZulu name “Uvo Lwakho”, meaning “your perception”, was selected after much discussion, including with study community members, as it portrayed the essence of the research project: an exploration of community perceptions of the place they lived.

## Methods

A rapid qualitative assessment approach was used to collect data in selected communities with a particular focus on perceptions and experiences of HIV infection risk, prevention, treatment and care options. The objective was to identify structural components and aspects of a community, including the culture and environment which contribute to HIV and TB infection to support development of targeted infection prevention interventions. This methodological approach was based on a range of participatory research techniques adapted from previous research in Zambia and South Africa (Bond, Chiti et al., 2016; Bond, Hoddinott et al., 2016) and used qualitative study techniques including community mapping, group discussions, observations, and key informant interviews (KIIs) (Kielmann, Cataldo, & Seeley, 2011).

### Setting

The study was conducted by the Africa Health Research Institute (AHRI), in UMkhanyakude district, KwaZulu-Natal, one of the poorest areas in South Africa with high HIV prevalence (Bärnighausen et al., 2008; Tanser et al., 2014; Welz et al., 2007) The sample comprised community members from four places purposively selected to represent areas with documented high prevalence of HIV (A and C) and with low prevalence (B and D).

### Community entry

The study was presented to the Community Advisory Board of AHRI. This board is made up of members of the tribal and civil councils in the local UMkhanyakude district. Their advice was sought to determine language and terminology acceptable to members of the community and how to build trust with the stakeholders we were working with. This was followed by an introduction of the study in the communities by the AHRI community engagement unit. This involved meetings to seek permission to work in the area and then community meetings and roadshows to explain the purpose of the study, giving members the opportunity to seek clarifications and get answers to any questions or concerns.

### Data collection

A pilot of all the activities was conducted between November and December 2015, and the selected methods were reviewed and data collection tools revised.

A team of four members, two men and two women, conducted data collection at each site over 12–15 days in late 2016, using the techniques detailed in Table 1.

### Spiral (a circular transect) walk

Before data collection at each place a spiral walk was conducted. This is a systematic walk, moving from roughly the centre to the outskirts of the area with the intention of getting an idea of the layout and conducting informal conversations with people met *en route*, to introduce the study. Immediately after the walk the team members recorded their walking route and documented their experience in field notes. The walks assisted the team to identify potential interviewees and places to conduct structured observations.

### Group discussions

A total of 22 discussions were held across the sites. Each group discussed their definitions of their community and perceptions of the experiences of HIV and TB prevention, treatment and care options. The discussion focused mostly on the impact that the geographical area and boundaries had on HIV and TB and perceived risks and associated vulnerabilities. Some participatory methods were used during the discussions to encourage people to talk: drawing maps of key features in the area, developing pictures which represented the type of place it was and developing lists of HIV-prevention options or risks and sorting into level of importance.

### In-depth and key informant interviews

These were interviews with key informants selected in consultation with community members, representing different types of people living in the place. The interviews were structured into three sections covering, for each place: knowledge of HIV and TB risk, awareness of different prevention methods, information on access and availability of treatment and care options; history of HIV and TB testing in the community and the extent of ART and HIV stigma and impact; and how they explain different rates of HIV and TB.

### Observations

The research team carried out structured single observations at different places and times of the day. This was conducted systematically with explicitly formulated rules on what the research team should look for and how they should record it, using an observation schedule. The focus was to observe mobility within specific areas, for example, bus station, social grant pay point, and tavern. We also observed behaviours, activities and the demographic characteristics of people entering the place. Direct observation has the advantage of recording activities as they happen and not only relying on self-reported behaviour (Kawulich, 2005). This method was useful in providing an overview of the different places people interacted in the community often described by participants in the other data collection activities.

### Ethical considerations

The study was approved by the University of KwaZulu-Natal Biomedical Research Ethics Committee (BE197/15). Written informed consent was taken from all participants before they engaged in an activity. At the end of each set of community fieldwork activities, ethical considerations were discussed, reflected on and documented by the research team to ensure that ethical and governance guidelines were adhered to, and to identify any issues that may need to be addressed. No names of informants were recorded; instead pseudonyms were used and names of organisations anonymised. The research team members were trained to work with particular vulnerable groups (such as people living with HIV who had suffered stigmatisation) and equipped with resources to help them signpost and refer participants to necessary counselling and treatment services available in the community.

### Data analysis

Debriefings with the team were held regularly throughout the data collection period to review progress on recruitment against the sampling frame and to discuss emerging themes from the data. Data were transcribed and translated from isiZulu to English by the team and quality checked by the team leader. Data analysis was conducted manually using aspects of theoretical propositions case study strategy (Yin, 2009). Data from each community were analysed as a case and treated as a separate data set. Grounded Theory principles were used in the thematic analysis of the data (Strauss, 1987). Initial steps involved full data familiarisation to understand the vast data available, followed by coding based on indicators of categories in events and behaviour. The first two transcripts were coded by four members of the team to develop a coding frame and to ensure inter-rater reliability (Pope, 2000). The coding frame with description of each code was reviewed by the lead analyst (the first author) and any changes or suggestions were discussed with the team. Each team member then applied the coding frame to all the data for their respective community. Weekly team meetings were held to discuss consistency and meaning of the codes and developm new codes that were not in the coding frame. These codes were later reviewed by the team and classified into categories. Identification of themes, coding and charting were conducted simultaneously, comparing codes both within and across sites.

## Results

### Study population and sites

The final sample consisted of 230 individuals (*n* = 57 in Site A; *n* = 55 in Site B; *n* = 61 in Site C; *n* = 57 in Site D) all aged 19 years and over. Of the 230 individuals who participated in the study, 27 self-identified as a person living with HIV (PLWH). Demographic characteristics of the participants by data collection methodology are shown in Table 2.

Site A was the most densely populated site covering a total of 0.65 km^2^, with 209 housing compounds. It had access to a main national road bringing in visitors and therefore high levels of movement in and out of the community. The site had access to a 24-hour petrol station, shops, restaurants, and a late-night bar. The community had piped water supply delivered to some houses, however, most people collected water from unreliable community taps. No schools or health clinics were available within the community, and people travelled to nearby areas to access education and government clinics.

Site B was a quiet rural community, sparsely populated with scattered homesteads over 2.28 km^2^ and 54 housing compounds. Most community members owned livestock and large grazing fields. There were few older men in the area. People said they had died. The community had one grocery store, one primary school, a restaurant/bar frequented by most members of the community, and a nearby coal mine and game reserve, which provide most (limited) employment opportunities. People in the community know each other by name with amaZulu traditional practices such as the Reed dance, a coming of age ceremony for unmarried girls, reported to take place. Community members reported that water services were still in the installation process, and they accessed clinics outside the area.

Site C was a semi-rural area with a surface area of 1.93 km^2^ and 172 housing compounds, with a mixture of traditional “rondavel” (round huts) housing and more modern brick houses. There was a significant rental community with many local businesses and private residences rented by non-locals and foreign nationals. Water and electricity access varied according to income, with some households unable to afford water tanks and using the river and community taps as a water supply. At the time of the study there was a clinical research trial clinic in this area which closed 3 months after data collection took place.

Site D was a rural community with a surface area of 4.31 km^2^ and 82 housing compounds, surrounded by rivers and hills with limited basic infrastructure provision, including access to electricity and water via community taps. No schools were available in the community, and children walked around 4 km each way to access primary and secondary education. There was one grocery shop and a government social grant access point. Twice a month the community had access to a mobile health clinic, but had to travel to nearby clinics for other health needs and emergency care.

### Local perceptions and definitions of community and their symbolic boundaries

From the observations, the researchers reported notable differences in community dynamics in areas with high and low-density housing. Sites A and C were high density areas, located at a walkable distance to the local town, that provided access to jobs or the opportunity to seek employment.

In Sites A and C members felt that their communities were overcrowded due to high levels of both internal and international migration for employment purposes. These sites had high levels of unemployment and very few income-earning opportunities, even though people continue to move there hoping to find work. This internal and cross-border migration was viewed negatively, especially by younger men who saw foreign people as the cause for their poverty, as one participant expressed: “*They are foreigners and we hate them they are just renting our territory. For them to take away our economy status and our women*” [group discussion (GD) with younger men, Site C].

According to community members, having money distinguished between different classes of people, with the wealthier people having bigger houses with more space, and better food and poor people unable to afford basic needs:

I sleep outside under the stars because I cannot go to someone’s house…there is nothing we are assisted with, we are hungry and no one will offer maize meal to cook. This leads to not trusting each other [GD with older men, Site C].

This lifestyle has decreased pro-social behaviour among community members with other people ashamed to ask for help. Participants described how this has led to less trust among people in the community and affected relationships and interactions with one another instead of working together to improve their situation:

Because the thing that makes me fear to go to the neighbour and ask for maize meal is that the neighbour would say it is because of me that she sleeps hungry…that’s what makes us fear each other, if we can develop each other then that is when things will be ok [GD with older women, Site A].

The divide between the rich and poor affects social cohesion. Interviewees alluded to the fact that the variation in wealth had changed the way of life and the social interaction within the community. Social networks were weakened and people no longer helped one another, as this young woman stated:

No I think that this community of [C] is not collaborated/united, to everyone youth and adults, there is no unit. Because I can get into trouble here with someone young next to me he/she will not help me … Even older people, there is no collaboration [younger woman, Site C].

… we as neighbours we don’t eat what you have, yes, we discriminate each other amongst us [female, PLWH, Site C].

In contrast, although there was indication of poverty, respondents from sites B and D described a sense of mutual support and more community cohesion. Although they experience high levels of unemployment that force men and young people to go to the nearby towns, community members still have what they described as “ubuntu” ,an African philosophy of showing humanity to one another, including helping one another when in need as the extracts below show:

Eh I am saying that because maybe let us say something has happened, to one of the neighbour … people here are able to quickly support that person [interview with a female, Site D].

I think this community we are residing in is a united rural community, not like township community. In other places, we know that you cannot borrow something from your neighbours to make living but here in farms we borrow [GD PLWH, Site B].

### Local insights of HIV and TB “hotspots” and drivers of infection

More participants from sites A and C indicated their community had a high rate of TB and HIV infection. There was the perception that they were aware the community had many people who were ill, as one participant described:

There are diseases like HIV, TB and many more diseases but its HIV and TB that I regard as top ones I think there is cancer maybe people are still not brave to do check-up for such diseases [GD with older women, Site A].

Participants felt that some places had higher risk due to their proximity to the towns or main roads where people had easy access to restaurants or hotels and alcohol. Restaurants were used to lure young girls who might be hungry and provided entertainment:

Here at pic and pay it is where they prostitute themselves, there is a hotel inside… there are these brothers who work in the road … brother pays for you entry, and do the ordering, and the thing you know…and pay you at month end [GD with older women, Site A].

Young women in Site B could identify the various places where people engaged in risky sexual behaviours which was mostly through sex work. They described where sex workers went to find clients and who some of these clients were. They also expressed how some people left the community and only returned when they were sick:

Others work as sex workers (outside the community) … Others they hike trucks there are beds behind driver seats… Others they seat next to the light of Somkhele Mine… In most cases they come back after 3 months others don’t come back at all whereas others come back once they are sick [GD with younger women, Site B].

In other communities, they talked about abstinence and encouraged certain practices such as “ukusoma”, which they called thigh sex, a common Zulu practice (which avoids penetrative intercourse). This was discussed as a potential preventative method. However, there was discussion of some incorrect methods such as washing after intercourse.

Community members described how issues such as lack of employment led to young people engaging in health-related risk behaviours including substance abuse with an increase in females using alcohol. Others engaged in risky sexual behaviour such as transactional sex.

I will say everybody because also females are now drinking. You see the manner they do that they are no longer respecting themselves. Also, youth there are those who are deep in alcohol [KII, Site D]

Members of the community viewed alcohol and other substance abuse as a driver of the HIV epidemic, as in most cases this can lead to intoxication and having multiple sexual partners. Participants said lack of access and availability of protective methods such as condoms contributed to HIV transmission:

They are high risk for poor people getting infection as they are often sleeping there are few chances of protecting themselves as there are no condoms [GD with younger women, Site B].

Poverty itself was also described as a driver of the HIV epidemic, as it increased the vulnerability of some young girls to infection. Due to the social divide described in the symbolic boundaries of rich, poor and middle class, poor young people were engaging in a form of transactional sex, and non-use of condoms:

By that time, you no longer care about your life, because you have been through a lot of difficulties in life so having this person as part of your life is better because she has money so you will sleep with her without a condom [GD with younger men, Site D].

The term transactional sex was, however, not used by some respondents even though they described how young people may expect material goods in exchange for a sexual relationship. There was a perception that in most relationships couples exchange gifts or monetary value and therefore it was better to be in a relationship with a richer person whose gifts would have bigger monetary value.

### Barriers to the uptake of prevention and treatment of HIV and TB

Stigma and fear associated with being HIV-positive was one of the reasons people in the community did not want to know their HIV sero-status. People were reluctant to get tested, especially men:

Men are not really used to going to the clinic. When they go it is because they are forced and when he feels better he leaves the medication [female, KII, Site C].

Participants described how the setup of the clinic stopped some people from going to receive their treatment. They felt that the separation of services where TB and HIV patients use separate facilities and have specific files that are visible to everyone perpetuates the problem of gossip and stigmatisation:

I saw a girl running away at the clinic. She said to me please carry this file for me because that woman will gossip about me in my neighbourhood. This thing is very hurtful because another person will be scared until she dies [Female, PLWH in Site A].

Participants also described what can be observed as a desensitisation to HIV. There was an awareness of the HIV epidemic and increase in infection in their communities as well as availability and accessibility of antiretroviral therapy (ART). However, there was a sense from participants that HIV was like a common ailment:

HIV is like having flu… no one is negative [older woman, Site A].

To some it has even become a saying, like when you greet them they will respond like “we are fine just the virus that I walk with [KII, Site B].

This contrasts with some PLWH who described how they are still stigmatised and do not want to disclose their status. It seems that some participants viewed HIV as being unavoidable as they were either infected or affected by the disease. They described a sense of helplessness in that people in the community do not have a perceived sense of control or power over the HIV epidemic. This lack of power led to a certain vulnerability to infection through negligent behaviour despite knowledge of HIV prevention among community members, especially with the young people. One researcher describes an interaction with young men during an observation at the community tap in Site A: “*When asked if they are not worried about being infected with HIV, the young men laughed and agreed on saying that everybody is now HIV positive in South Africa, so using the condom or not is now the same*”.

When it came to TB, some respondents could describe preventative measures, however, there was limited knowledge of TB in general in the community. A variety of perspectives were shared on TB infection. However, there was more focus on HIV than on TB. Other respondents shared how they did not know much about TB transmission and prevention:

The problem is that I don’t even know how TB is contracted… [GD with young women, Site A].

I don’t understand TB because they say it is transmitted through air so I don’t know how it can be prevented because you cannot live without inhaling [KII, Site B].

Possible modes of TB transmission mentioned by the respondents included poor diet, sharing drinking bottles at the tavern, being in public places, and not having open windows in homes. When asked about preventative measures the responses included covering coughs and eating well.

Other barriers to prevention and treatment were due to what was described as unequal power relations. Some participants expressed that the people may be controlled by the other person in a relationship:

No, they are controlled by the males. A person would say I cannot be with you anymore if you are going to proceed with these tablets for the virus and because you love him very much, more than your life, you see when you are leaning against the wall and the wind blows it and it falls you will also fall [female, PLWH, Site A].

This power imbalance was usually with the male being the dominant partner and the female having less power to make decisions for her health.

## Discussion and conclusions

The findings of this study suggest that for these communities (in areas of high and low HIV prevalence), health and the HIV and TB epidemic are less of a concern than socio-economic deprivation. Although places promoting high risk behaviour were easily identified by community members, they had a more nuanced understanding of risk, and local descriptions of vulnerability were less about geographic boundaries and more about behaviours, described in wealth and social terms.

The results of this study suggest that many people may leave the rural areas in search of economic independence through seeking employment, and to live closer to amenities such as schools, health facilities and other facilities that more urban areas may offer. Previous research has shown that rural to urban migration has increased as people seek economic independence, and migration is associated with high sexual risks (Li et al., 2004; Lurie et al., 2003; Sambisa & Stokes, 2006). In our study, participants were aware of the health risks associated with living in these urban and peri-urban areas. Participants in these high density urban areas described negative features of these areas such as lack of community cohesion and lack of employment, and yet they still preferred to be there for economic reasons. Participants were also able to identify places they described as having high HIV prevalence where people engage in risky behaviours. However, this knowledge and awareness did not change their desire to live there. This is consistent with literature that shows a weak relation between knowledge and behaviour, instead showing that behaviour is influenced more by aspects such as norms and perceived sense of control (Mnguni, Abrie, & Ebersohn, 2016; Olley, 2003). Community members were more concerned with other realities of life such as lack of employment opportunities, seeking economic stability and preservation of social networks.

The results from the four sites showed that the extent of inequity of access to health, and educational resources in these rural areas in South Africa is still problematic and widespread. Key informants identified different aspects and dimensions of lack of access as well as limited opportunities which stifled people’s choices and sense of control. These findings contribute to previous literature which shows that socio-economic deprivation plays a major role in the spread of both HIV and TB infections (Dawson, 2013; Dube, Benhildah, Marshall, & Ryan, 2016; Ransome, Kawachi, Braunstein, & Nash, 2016). It is essential to consider people’s priorities when developing interventions to increase the probability of acceptance and effectiveness of solutions proposed.

The declining economy heightens the conditions that increase risky sexual behaviours. The findings in the study indicate that people may be vulnerable to HIV infection because of lack of economic independence and a decreased sense of control over their lives to change behaviours. The social vulnerabilities experienced by communities may force individuals to move to more urban areas with aspirations of gaining economic independence. This move, however, does not necessarily fulfil those aspirations leaving people more despondent with no employment opportunities, and less community support, no income and limited access to resources. This was evident in tour study were some individuals had less access compared to others based on where they lived and their perceived wealth. The disparity between the wealthy and the poor in the communities was very clear and described as a contributory factor to the increase in HIV and TB infection. Although poverty has been identified as one of the drivers for the HIV transmission through increased transactional sex (Cluver, Orkin, Boyes, Gardner, & Meinck, 2011; Cluver, Orkin, Yakubovich, & Sherr, 2016; Steinert, Cluver, Melendez-Torres, & Herrero Romero, 2016), findings from this study show that it may also be a contributory factor to migration decisions with young people moving to places where they engage in risky behaviours. Although there seems to be increased practice of risky behaviours in urban than in rural areas, as shown in the comparison of the four settings in this study, the findings also indicate that the context within which a person lives has an influence on their behaviour and these larger social processes contribute to their perceived control in positive behaviour. The findings, however, indicate that in this generalised epidemic setting, the lack of opportunities including employment and inequitable access to resources may increase people’s risk of HIV infection. Regardless of their residential settings, the socio-economic factors play a larger role in activities that promote HIV transmission. This is consistent with previous research that states that HIV transmission is more about what people do and their social vulnerabilities (Guzman, 2001; Knox, Sandfort, Yi, Reddy, & Maimane, 2011; Mann & Tarantola, 1996). The association between HIV and migration is not only due to infection after but also before migration, as Anglewicz, VanLandingham, Manda-Taylor and Kohler (2016) showed how HIV-positive individuals are more likely to migrate and leave their village/home than those who are HIV-negative. Some people may move closer to clinics or away from where they feel they will experience stigma.

**Figure 1:**
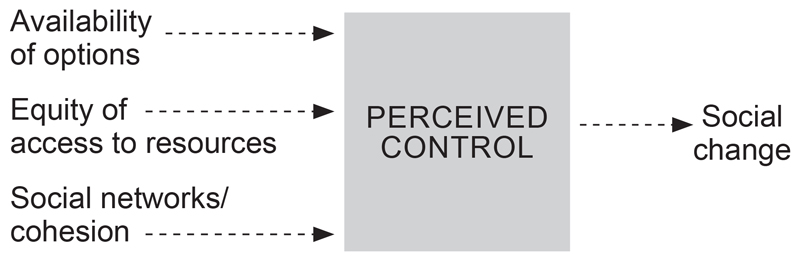
Postulated conceptual model illustrating dimensions of social change in addressing the HIV and TB epidemic

This combination of findings provides some support for the conceptual model proposed (Figure 1) on the mechanisms of perceived sense of control as a way of promoting social change.

Researchers have acknowledged and given evidence that empowerment is a necessary step in behavioural change, especially within public health. However, it is important to elucidate some of the dimensions of daily life and principles that anchor relationships between perceived sense of control and empowerment. Our conceptual model posits that cohesion, equity and the availability of opportunities are mechanisms that lead to a perceived sense of control. These dimensions should be carefully considered and incorporated in developing structural interventions to address the HIV and TB epidemic in rural settings. A structural approach might emphasise a focus on the broader effort of addressing social inequality and health equity as indicated by the earlier work of Friedman and McKnight (2003). Addressing these proximal issues such as increasing availability of opportunities (i.e., employment, cash transfers and recreational): equity of access to resources (i.e., increased frequency of mobile clinics and ensuring affordable easy access to protection such as condoms); and programmes that promote cohesion (i.e., training and supporting healthcare providers in communication and confidential care,) will in the long run influence social change.

Community cohesion and empowerment theories suggest that perceptions of control are important because they motivate people to develop community-focused problem-solving strategies to effect positive change (Dahlgren & Whitehead, 2007; Whitehead et al., 2016). Sen’s (1999) theories articulate that the availability of opportunities should be matched with capability in order for people to access those opportunities/resources. This lack of ability or perceived control underpins socio-economic inequality.

Another important finding is the perception that some young people are no longer concerned about contracting HIV as they felt that it is now a common disease. This finding suggests that even now, some people may feel powerless and have a fatalistic view of HIV which can affect prevention behaviours (Hess & Mbavu, 2010; Meyer-Weitz, 2005) and increase the epidemic. This is despite the wide roll out of ARV programmes and a “treatment as prevention” trial ongoing in one of the communities. The community was more aware of the availability and accessibility of a mobile clinic service offered by the trial and less about ART as a preventative method. This has implications for future programmes and improving people’s knowledge of the prophylactic aspect of treatment.

Interventions should initially focus on the broader structural issues that influence behaviour within those geographical areas identified as needing targeted preventative resources (Friedman, 2001). These results show that high risk behaviours function within a social context. A change in these social and economic structures and processes is needed for effective HIV prevention, as these realities have a significant influence on risk behaviours. Broader cultural factors should be considered based on African socio-cultural perspectives to reduce social alienation and disintegration which can help communities face the daily challenges they have in addressing the epidemic and improve sustainability of solutions (Meyer-Weitz, 2005; Mkhize, 2004).

Increasing the resources directed towards those with lower access on its own does not constitute a solution, as individuals may not feel that they have the capability of accessing and utilising the resources. It is important to continue raising awareness of both HIV and TB, however, this study suggests we need to iteratively adapt interventions to the social context, and the specific vulnerabilities and improve the communities’ sense of control. Interventions need to move beyond the simple allocation of resources to those identified as needy as this may not necessarily be sustainable and does not promote the long-term behavioural change needed to eradicate disease transmission and increased infection. Rather this study suggests that perceived sense of control is an important principle for sustainable interventions that may empower communities and promotes access and utilisation of resources.

## Figures and Tables

**Table 1:** Summary of the activities and their associated research questions and methods

Research activity	Key research questions	Methods
Spiral walk	What are the activities and movement (short distance travel, commuting and longer migration patterns) of different age and gender groups? Where are the places of significance to HIV and TB transmission and prevention in the community?	Transect spiral walk starting from central point, moving in concentric circles around the community and taking care to stop, listen, look and chat on the way. GPS coordinates and observation notes, using a simple checklist (developed during the training).
Observations: shops, significant events, weekend venues, entry/exit points, meeting spots in residential areas	What is happening in this community linked to: HIV; risk-taking; economic activity; mobility, TB transmission	Observations carried out by researchers at different times and places in the community (choice of location based on information collected during previous activities). Activity reports and checklists used to record details.
All focus group discussions	What kind of community is this? What are the local boundaries in this place? What is happening in the community in terms of HIV? Where are the HIV and TB hotspots?	** “What kind of place is this”:** participants describe their community using pictures/words. **Mapping:** social map showing local boundaries, places of significance to different groups of people (meeting places, main residential areas, etc.) and that identifies specific HIV and TB hotspots. **Wealth/wellbeing, poverty and risk-taking:** participants describe different types of people in the community and explore influences on risk-taking.
1) Healthcare providers discussion	What are community and individual perceptions of and experiences with HIV prevention, treatment and care options and TB care and control?	**What kind of place is this?** (as above) **Concept mapping:** participants discuss and produce unlimited ideas and concepts linked to HIV and TB prevention in their community.
2) Community group discussion	What are local perceptions of and experiences with HIV and TB prevention, treatment and care options? How do wealth and poverty have an impact on HIV risk-taking and other behaviours and risks associated with TB disease? What are the main practices/causes that lead to HIV infection and TB disease?	**What kind of place is this?** (as above) **Idea sorting and sharing:** participants sort ideas into piles and then rank them (i.e., what is high risk/low risk) **Institutional mapping:** participants identify institutions involved in HIV service provision in the community. They share perceptions of relationships between the institutions and identify the most important. **Wealth/wellbeing, poverty and risk-taking** (as above)
3) People living (openly) with HIV group discussion	What is the experience with HIV testing and ART in each community site? What is their knowledge of HIV services, including treatment? What is their knowledge of TB services?	**What kind of place is this?** **History of HIV testing & ARVs timeline:** participants share their knowledge and experiences of HIV testing, ART, and treatment, including as prevention. Key events in the local history of ART are recorded on a timeline. **History of TB and other co-morbidities:** Participants share their knowledge and experiences of TB, hypertension and any other conditions they mention.
In-depth and key informant interviews	What are the HIV and TB risk, prevention, treatment and care options & stakeholders in this place? What is the history of HIV and TB testing and ART and HIV stigma in this place? How might they explain different rates of HIV and TB (and perhaps hypertension, disability, etc. if these are perceived as being linked, for example) in different places (or do they assume uniform risk)?	**Semi-structured interview** covering key themes

**Table 2:** Summary of study population per site

Sites	Focus group discussions (GDs)	In-depth interviews (IDIs)	Key informant interviews (KIIs)	Observations
Community A	8 young men 18–35 years 7 young women 23–30 years 8 older women 35–65 years 7 PLWH 33–43 years 5 young women 19–25 years 7 older men 36–66 years 7 people engaged in HIV care 35–70 years	8 IDIs 35–65 years [4 × traditional healers (1F, 3M) 1 × former caregiver (F) 2 X PLWH (F) 1 × traditional and faith healer (F)]		Transect spiral walk Petrol filling station 2 bars Church Community water tap
Community B	12 young men 19–33 years 6 people engaged in HIV care 45–58 years 8 young women 19–34 years 10 older women 35–60 years 8 PLWH 19–54 years 4 older men 45–62 years	3 IDIs 30–71 years [1 × retired caregiver (F) 1 × pastor (M) 1 × Community Advisory Board member (F)]	4 KIIs 33–114 years [1 × shop/bar attendant (F) 1 × retired ward councillor (M) 2 × teachers (F)]	Transect spiral walk Bar Community entry/exit point (public transport taxi stop)
Community C	9 young men 21–35 years 10 older women 40–64 years 10 young women 18–28 years 8 people engaged in HIV care 23–45 years 9 older men 36–79 years	10 IDIs 19–65 years [8 × PLWH (8F, 2M) 1 × HIV Counsellor (F) 1 × Nurse (M)]	5 KIIs 23–80 years [1 × shop owner (M) 1 × bar owner (F) 1 × tribal policeman (M) 1 × snuff seller (F) (herbal tobacco) 1 × herbalist (specialising in STI treatment) (M)]	Transect spiral walk Shop Sports bar Faith healing place Church
Community D	12 young women 19–35 years 13 young men 19–35 years 12 older women 36–89 years 11 older men 36–95 years	4 IDIs 27–68 years [2 × PLWH (F) 1 × caregiver (F) 1 × Community Advisory Board member (F)]	5 KIIs 24–68 years [1 × shop attendant (F) 1 × pastor (M) 1 × pastor’s wife (F) 1 × sports coach (M) 1 × teacher (F)]	Transect spiral walk Shop Mobile clinic Pension paypoint Nazareth Baptist Church
